# How SARS-CoV-2 (COVID-19) spreads within infected hosts — what we know so far

**DOI:** 10.1042/ETLS20200165

**Published:** 2020-12-03

**Authors:** Sumana Sanyal

**Affiliations:** Sir William Dunn School of Pathology, University of Oxford, South Parks Road, Oxford OX1 3RE, U.K.

**Keywords:** coronaviruses, immune subversion, intracellular lifecycle, pathogenesis, SARS-CoV-2

## Abstract

Severe acute respiratory syndrome coronavirus 2 (SARS-CoV-2), the causative agent of the ongoing pandemic of coronavirus disease 2019 (COVID-19), belongs to the betacoronavirus genus and shares high homology to the severe acute respiratory syndrome coronavirus (SARS-CoV) that emerged in 2003. These are highly transmissible and pathogenic viruses which very likely originated in bats. SARS-CoV-2 uses the same receptor, angiotensin-converting enzyme 2 (ACE2) as SARS-CoV, and spreads primarily through the respiratory tract. Although several trials for vaccine development are currently underway, investigations into the virology of SARS-CoV-2 to understand the fundamental biology of the infectious cycle and the associated immunopathology underlying the clinical manifestations of COVID-19 are crucial for identification and rational design of effective therapies. This review provides an overview of how SARS-CoV-2 infects and spreads within human hosts with specific emphasis on key aspects of its lifecycle, tropism and immunopathological features.

## Introduction

Coronavirus disease 19 (COVID-19) is a new and highly transmissible viral infection caused by the severe acute respiratory syndrome coronavirus 2 (SARS-CoV-2). Genomic analyses have revealed that SARS-CoV-2 is phylogenetically closely related to severe acute respiratory syndrome-like (SARS-like) bat viruses, implicating bats as a possible primary reservoir [1]. Although an intermediate animal source has been speculated to be involved in its species jump, the identity and mechanism of transfer to humans is not known. Nevertheless, the rapid human to human transfer indicates a high transmission potential of this virus [2].

Clinical manifestations in COVID-19 patients indicate highly varied disease pathologies of SARS-CoV-2 infection in humans. The virus passes through the nasal and larynx mucosa and enters into the lungs through the respiratory tract. Fever and cough are among the early and most common symptoms of infection [3]. SARS-CoV-2 can potentially enter the peripheral blood from the lungs and spread into cells in tissues that express angiotensin-converting enzyme 2 (ACE2), such as the lungs, heart, renal and gastrointestinal tract [4]. The median time from symptom onset to acute respiratory distress syndrome is about 8 days [5]. Infection is accompanied by moderately low white blood cell count in peripheral blood and mild lymphopenia in the early stages of infection becoming more significant in severe cases [3,5]. Although not well-documented, current evidence indicates that B lymphocyte reduction may also occur early in the disease, which may, therefore, affect antibody production in the infected host. In severe cases, lymphocytes are significantly reduced; however, the mechanism of this dramatic reduction in lymphocytes remains unclear. Besides, pro-inflammatory factors such as IL-6, IL-10 and TNFα are significantly increased, often referred to as a ‘cytokine storm’, which also contributes to disease aggravation approximately 7–14 days after onset [6–8]. Based on the features of disease presentation, the clinical course of SARS-CoV-2 has been proposed to be broadly divided into three phases: the viremia phase, the acute phase (pneumonia) and severe or recovery phase [9]. The immune function of the infected hosts appear to be the primary determinant of disease outcomes. Without any underlying comorbidities the infection can often resolve even at the acute phase. However, in older patients or in those with impaired immunity, combined with other comorbidities such as hypertension and diabetes, the disease often progresses into severe or critical forms [10].

Most known human coronaviruses cause the common cold. This family of viruses had, therefore, remained relatively obscure, with limited numbers of severe human diseases attributed to them. However, with the emergence of a new human coronavirus responsible for severe acute respiratory syndrome in 2003, coronaviruses became much more recognized. With the occurrence of the SARS and the MERS outbreaks within the past couple of decades combined with the ongoing pandemic, coronaviruses are now considered ‘emerging pathogens.’ The origin of the SARS-CoV-2 poses interesting questions about coronavirus evolution and species specificity: in particular the role of reservoir species, the role of recombination and divergence date. Here, we discuss what is known about the infection cycle and spread of this virus once an individual is infected. It has become evident that although the body of information gathered over the last 30 years regarding coronavirus replication and pathogenesis has helped us understand the biology of SARS-CoV-2, a lot more work needs to be done for the control and prevention of this class of coronaviruses.

## Entry mechanisms

Coronaviruses are enveloped, positive-sense RNA viruses that replicate in the cytoplasm. Attachment and entry into susceptible host cells are essential components of viral spread — from tissue tropism to cross-species transmission — especially for the betacoronaviruses. The genome of SARS-CoV-2 follows the characteristics of known coronaviruses [2]. The 5′ two-thirds of the genome encodes for 16 non-structural proteins, whereas the remaining one-third encodes the structural proteins Spike (S), Envelope (E), Membrane (M) and Nucleocapsid (N). All coronaviruses encode a surface glycoprotein, Spike, which binds to the host cell receptor and mediates viral entry. Fusion of their envelope with the host cell membrane usually at the acidic endosomal compartments or less frequently at the plasma membrane results in delivery of their nucleocapsid into the host cell cytoplasm. The Spike glycoprotein (S) drives virus entry and is the primary viral determinant of cellular tropism. It is a class I fusion protein, and is critical for binding to the relevant receptor on the host cell surface as well as for mediating fusion between the host and viral membranes in a process driven by significant conformational changes in the Spike protein. For betacoronaviruses, the receptor-binding domain (RBD) region in the Spike protein mediates the interaction with the host cellular receptor. Binding to the receptor is followed by cleavage of the Spike by a proximal host protease, releasing the spike fusion peptide to facilitate virus entry. Known host receptors for betacoronaviruses include ACE2 for SARS-CoV and dipeptidyl peptidase-4 (DPP4) for MERS-CoV [11,12]. Previous studies have reported that RBDs from the lineage B of beta coronaviruses can be categorized into functionally distinct clades. Those from clade 1, which includes SARS-CoV-2 can enter cells expressing ACE2 [4]. This has been experimentally validated by several studies demonstrating the crystal structure of the RBD of the Spike protein with that of ACE2 [13,14]. ACE2 is enriched in the ciliated bronchial epithelial cells, which appear to be major targets of SARS-CoV 1 and 2 [4,11], whereas DPP4 is enriched in the unciliated epithelial cells, which serve as target cells for MERS infection [12]. Both receptors are expressed in the type II pneumocytes, which in turn are infected by both viruses. Apart from the ACE2 receptor, Neuropilin 1 has been recently identified as entry factor that function in concert with ACE2 to facilitate SARS-CoV-2 entry [15,16]. Notwithstanding, expression of ACE2 in combination with a host transmembrane serine protease has been shown to confer susceptibility to SARS-CoV-2.

**Figure 1.**
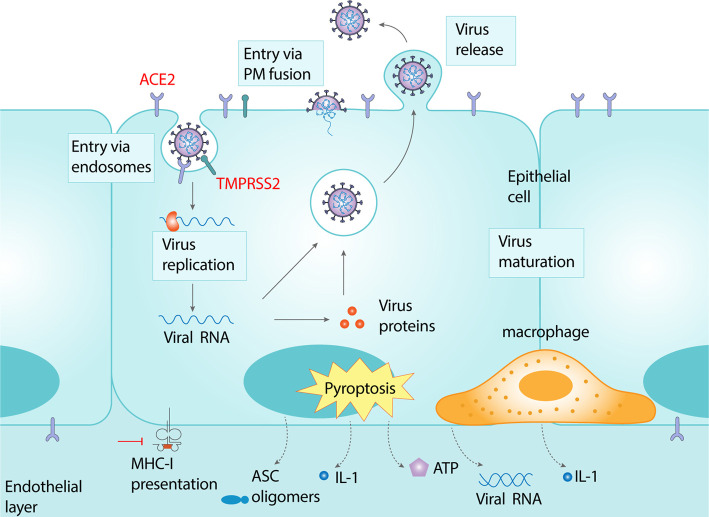
Schematic of the intracellular lifecycle of SARS-CoV-2 and associated immunopathology. Severe acute respiratory syndrome coronavirus 2 (SARS-CoV-2) infects cells expressing the surface receptors angiotensin-converting enzyme 2 (ACE2) and TMPRSS2, resulting in entry of the virus via the endocytic machinery or upon fusion at the plasma membrane. The viral genome is released into the cytosol upon fusion of the viral and host membranes and undergoes replication, transcription, translation and assembly to form viral progenies that are released into the extracellular space via unknown mechanisms. Amplification and release of the virus leads to host cell pyroptosis and release of damage-associated molecular patterns, including ATP, nucleic acids and ASC oligomers. This is accompanied by secretion of pro-inflammatory cytokines and chemokines culminating in a cytokine storm. On the other hand MHC-I restricted antigen presentation is downregulated most likely by binding of the viral Orf8 protein, resulting in attenuated T-cell activation, thereby contributing to the common clinical feature of lymphopenia.

Similar to other coronaviruses, SARS-CoV-2 entry occurs via a multi-step process of cell surface attachment, receptor engagement, proteolytic cleavage and membrane fusion that involves several distinct domains on the Spike protein [17] (Figure 1). While the RBD-receptor interaction is the best characterized step in the sequential cascade necessary for viral entry, recent studies have highlighted an important role host proteases play in facilitating entry, processing coronavirus progenies, and also as potential species barriers [18,19]. Analogous to these studies, exogenous addition of trypsin was found to enhance entry of SARS-CoV-2 [4,20]. The most favored host protease candidate is the transmembrane serine protease Tmprss2, although other members of this family as well as certain cathepsins are believed to be involved [20]. While it has been shown that host proteases cleave the Spike protein to allow for downstream membrane fusion, additional evidence suggests that proteases may also act on the receptor to activate it [20,21]. The SARS-CoV-2 Spike protein also contains a stretch of multiple arginine residues forming a S1/S2 cleavage site not found in the other closely related animal coronaviruses [22]. Characterization of the role of this multibasic cleavage site in SARS-CoV-2 infection revealed that the S1/S2 site is cleaved by furin and furin-like cellular proteases. Proteolytic cleavage at this site was found to be essential for spike-mediated cell–cell fusion and entry into human lung cells. Optimizing the S1/S2 site increased cell–cell, but not virus–cell fusion, suggesting that the corresponding viral variants might exhibit increased cell–cell spread and potentially altered virulence. These results provide a basis for the increased transmissibility of SARS-CoV-2 and suggest that acquisition of a S1/S2 multibasic cleavage site was perhaps essential for SARS-CoV-2 infection of humans [23].

## Viral replication

SARS-CoV-2 uses an RNA-dependent RNA polymerase (RdRp) for the replication of its genome and the transcription of its genes. This RdRp complex is the target of nucleoside analog inhibitors, in particular remdesivir, which has been shown to inhibit the polymerase of multiple coronaviruses [24]. Cryo-electron microscopy of the SARS-CoV-2 RdRp in an active conformation has revealed that the complex comprises the viral non-structural proteins nsp12, nsp8 and nsp7, and more than two turns of RNA template-product duplex. Nsp12 is the catalytic subunit of the RdRp of SARS-CoV-2, whereas nsp8 and nsp7 serve as accessory subunits [25–27].

Like other positive-sense (+) RNA viruses, including hepatitis C virus (HCV), Dengue virus, Zika virus and polioviruses, coronaviruses share the feature of establishing specialized membranous replication organelles with unique lipid compositions to enable robust viral replication [28–31]. Depending on the groups of the viruses and the time course of the infection, the structure, composition, and formation of (+) RNA virus replication organelles appear to be varied and dynamic [32]. Emerging data for viral RNA synthesis suggest that these replication organelles are evolutionarily conserved and form an essential step in the early stages of the viral lifecycle. The replication organelles serve multiple purposes: first, they provide an optimal microenvironment specialized for the synthesis of viral RNA by concentrating viral components (RNA and proteins) and host factors (specific proteins and lipid species) required for viral RNA synthesis. Necessary host factors are actively recruited to appropriate membrane sites in and around replication organelles where they can interact with viral components within these microenvironments, thereby generating high local concentrations with less interference from host membrane protein traffic or the possibility of soluble factors diffusing away [29,33]. Second, viral replication very likely takes place in the inner membrane facing the cytosolic interior of the replication organelles. Therefore, replication intermediates (e.g. dsRNA) can be physically shielded from the host innate immune defenses such as pathogen recognition receptors that are present in the cytosol [34]. Finally, formation of replication organelles allows spatial orchestration of the different steps necessary for viral replication and assembly. Hence, membrane remodeling plays a crucial role in the (+) RNA virus lifecycle. (+) RNA virus-induced replication organelles can be classified into single-membrane spherules, double-membrane vesicles (DMVs), cubic membranes/membranous webs, and planar oligomeric arrays depending on their morphological features.

Both MERS-CoV and SARS-CoV have been reported to induce ER-derived DMVs [35,36]. In SARS-CoV-infected Vero E6 cells, DMVs that are distributed throughout the cytoplasm can be observed as early as 2 h post-infection (p.i.), with a diameter of 150–300 nm [35]. The number of DMVs increases dramatically at 4 h p.i., accompanied by their connection to ER structures [35]. A very recent report unveiled a molecular pore complex that spans both membranes of the DMVs induced by MHV and SARS-CoV-2 [37]. Sub-tomogram averaging of the DMV-spanning complexes revealed a 6-fold symmetry. A cytosolic structure with a crown-like morphology extends ∼13 nm into the cytosol and is based on a ∼24 nm wide platform embedded between the two layers of DMV membranes, which maintain the typical inter-membrane spacing found in DMVs. The complex towards the cytosol side has an opening of ∼2–3 nm, allowing the transition of RNA strands that later would be encapsidated in the cytosol [37].

The extensive membrane rearrangements induced during coronavirus replication necessitates that besides significantly enhanced lipid biosynthesis, lipid metabolic enzymes also regulate DMV formation. Cytosolic phospholipase A_2_α (cPLA_2_α) is a lipolytic enzyme that catalyzes the hydrolysis of membrane phospholipids at the sn2-position and release fatty acid, lysophospholipid (LPL) and arachidonic acid (AA). Several mass spectra-based lipidomic studies have shown selective up-regulation of downstream products of cPLA_2_α activation, including glycerophospholipids, LPL and fatty acid in MERS-CoV-infected or HCoV-229-infected cells [38,39], and long-chain polyunsaturated fatty acids (PUFA) in SARS-CoV-2 infected patient sera [40].

## Assembly, release and cell-to-cell spread

As with other coronaviruses, the mechanisms of assembly, release and spread of SARS-CoV-2 is one of the least understood processes. Previous studies have shown that expression of SARS-CoV structural proteins S, E, M and N expressed in Vero E6 cells can form virus like particles (VLPs) that are subsequently released into the culture medium. Packaging of viral RNA into such VLPs required the viral N protein and a packaging signal within a 579 nucleotide long domain of the genomic RNA [41]. The minimum requirements for VLP formation are under contention. Several studies have shown that formation of coronavirus VLPs depended on either M and E proteins or M and N proteins, whereas others have reported that both E and N proteins must be co-expressed with M protein for the efficient production and release of VLPs, suggesting that the mechanism of SARS-CoV assembly differs from that of other studied coronaviruses [42]. When co-expressed, the native envelope trimeric S glycoprotein is incorporated onto VLPs [42].

A very recent study has proposed an interesting model in which betacoronaviruses use the lysosomal pathway for egress from cells [43]. Intact coronaviruses had previously been detected in lysosomes but had not been characterized [44]. Egress routes of most enveloped RNA viruses have been ill-characterized and assumed to be either via the conventional secretory pathway or by budding at the plasma membrane. More recent evidence on flaviviruses have revealed that autophagosome-derived organelles can transport virus progenies to the extracellular space [45,46]. Coronavirus-containing lysosomes were found to be deacidified, resulting in disruption of lysosomal activity, antigen presentation and associated innate immune responses, therefore, providing a possible advantage to virus amplification [43]. Whether virus progenies are carried in these deacidified lysosomes into the extracellular space is yet to be characterized.

Although the underlying mechanisms of cell-to-cell spread of SARS-CoV-2 have not been identified so far, its cellular tropism has been characterized both *in vitro* and *in vivo* models. Tropism and replication of SARS-CoV-2 in *ex vivo* cultures of human bronchus, lung and conjunctiva revealed that SARS-CoV-2 was able to infect ciliated, mucus-secreting, and club cells of the bronchial epithelium, type 1 pneumocytes in the lung, and the conjunctival mucosa [47]. Replication competence of SARS-CoV-2 was found to be similar to MERS-CoV, higher than SARS-CoV, but lower than H1N1pdm in the bronchus. On the other hand, replication of SARS-CoV-2 was similar to SARS-CoV and H1N1pdm, but lower than MERS-CoV in lungs. In the conjunctiva, SARS-CoV-2 replicated more efficiently than SARS-CoV [47]. Additionally, in human small intestinal organoids (hSIOs), both SARS-CoV and SARS-CoV-2 were found to readily infected enterocytes, as measured by confocal and electron microscopy. Enterocytes supported virus replication and produced infectious viral progenies; mRNA expression analysis of hSIOs revealed that infection by these viruses triggered a generic viral response program [48]. These findings were corroborated in a separate study that reported productive infection of SARS-CoV-2 in ACE2+ mature enterocytes in human small intestinal enteroids. In this infection model, expression of two mucosa-specific serine proteases, TMPRSS2 and TMPRSS4, facilitated SARS-CoV-2 spike fusogenic activity and promoted virus spread within these cells [49].

## Evasion of host immunity

A major contributor to spread of infection within infected hosts is the ability of viruses to evade or subvert host innate and adaptive immune responses and spread from cell-to-cell within and between different tissues and organs. Aggressive inflammatory responses, host cell pyroptosis and release of damage-associated molecular patterns are implicated in the immunopathology of infection, leading to damaged lung airways (Figure 1). Clinical observations of SARS-CoV-2 infection include mild to severe lymphopenia, suppression of interferon production at early stages of infection and increased production of pro-inflammatory cytokines [50,51]. However, mechanisms adopted by SARS-CoV-2 to trigger host immune dysregulation and spread into different organs have not been well characterized. A preliminary study has shown that the viral protein encoding open reading frame 8 (ORF8), which shares the lowest sequence homology to SARS-CoV may contribute to increased pathogenesis of SARS-CoV-2. ORF8 was able to bind to MHC class I molecules and down-regulate their surface expression when transfected into HEK293T cells [52,53]. ORF8 co-localized with MHC class I molecules in lysosomes, indicating down-regulation of the latter via lysosomal degradation. Cytotoxic T lymphocytes (CTLs) from healthy human donors sensitized to the SARS-CoV-2 epitope SSp-1 were exposed to autologous dendritic cells pre-pulsed with SSp-1. ORF8-expressing HEK293T cells were more resistant to CTL-dependent killing compared with control cells indicating ORF8-dependent disruption of MHC-I restricted antigen presentation and subsequent T-cell activation [53]. These results were also replicated using CTLs isolated from a convalescent patient that responded to a mixture of SARS-CoV-2 N and S proteins. In a separate study, the papain-like protease (PLpro) was found to suppress interferon production and regulate SARS-CoV-2 spread [54]. Biochemical, structural and functional characterization of the SARS-CoV-2 PLpro revealed differences with SARS-CoV PLpro in regulation of host interferon and NFκB pathways. Although SARS-CoV-2 PLpro and SARS-CoV PLpro share 83% sequence identity they displayed different host substrate preferences: SARS-CoV-2 PLpro preferentially cleaved the ubiquitin-like interferon-stimulated gene 15 protein (ISG15) from proteins, whereas SARS-CoV PLpro predominantly targeted ubiquitin chains. In a separate study the de-ISGylating activity of SARS-CoV-2 PLpro was reported to trigger secretion of free ISG15 from infected cells which resulted in dysregulation of immune responses from monocytes and monocyte-derived macrophages [55,56].

This review provides a brief overview of the various mechanisms adopted by SARS-CoV-2 to amplify and spread within infected individuals. Collective evidence indicates that controlling the cytokine storm may be as important as targeting the virus. Therapies that inhibit various steps in virus lifecycle while triggering appropriate immune responses may become necessary to synergize and block disease progression. Along the same lines, although various efforts in vaccine development are currently underway, antibody dependent enhancement (ADE) is a general concern because mechanisms that underpin protective effects of antibodies have the potential to exacerbate infection or immunopathology as often seen in Dengue virus infections. The association between immune dysfunction, the possibility of ADE and outcomes of disease severity should, therefore, serve as a note of caution in the potential effectiveness of vaccines. A detailed investigation of the SARS-CoV-2 lifecycle, viral and host determinants of mild versus critical disease and a characterization of the interaction between the pathogen and immune cells is necessary to delineate the enhanced virus transmissibility and aberrant immune responses in SARS-CoV-2 infection.

## Summary

- The primary determinants of entry and cellular tropism for SARS-CoV-2 appear to be the ACE2 receptor and the TMPRSS2 serine protease. Cells expressing these two proteins have been uniformly found to be susceptible to SARS-CoV-2 infection.
- Disease progression in SARS-CoV-2 infection appears to be a combined effect of virus spread and aberrant immune responses.
- MHC-I restricted antigen presentation is down-regulated while pro-inflammatory cytokine secretion is up-regulated in SARS-CoV-2 infected cells resulting in lymphopenia and cytokine storms.

## Open Access

Open access for this article was enabled by the participation of University of Oxford in an all-inclusive *Read & Publish* pilot with Portland Press and the Biochemical Society under a transformative agreement with JISC.

## Abbreviations

ACE2: angiotensin-converting enzyme 2
ADE: antibody dependent enhancement
COVID-19: coronavirus disease 2019
CTLs: cytotoxic T lymphocytes
DMVs: double-membrane vesicles
DPP4: dipeptidyl peptidase-4
ISG15: interferon-stimulated gene 15 protein
LPL: lysophospholipid
ORF8: open reading frame 8
PLpro: papain-like protease
RBD: receptor-binding domain
RdRp: RNA-dependent RNA polymerase
SARS-CoV-2: syndrome coronavirus 2
